# Sex-specific relationship between non-alcoholic fatty liver disease and amyloid-β in cognitively unimpaired individuals

**DOI:** 10.3389/fnagi.2023.1277392

**Published:** 2023-10-13

**Authors:** Sung Hoon Kang, Heejin Yoo, Bo Kyoung Cheon, Jun Pyo Kim, Hyemin Jang, Hee Jin Kim, Mira Kang, Kyungmi Oh, Seong-Beom Koh, Duk L. Na, Yoosoo Chang, Sang Won Seo

**Affiliations:** ^1^Department of Neurology, Samsung Medical Center, Sungkyunkwan University School of Medicine, Seoul, Republic of Korea; ^2^Department of Neurology, Korea University Guro Hospital, Korea University College of Medicine, Seoul, Republic of Korea; ^3^Alzheimer’s Disease Convergence Research Center, Samsung Medical Center, Seoul, Republic of Korea; ^4^Department of Digital Health, SAIHST, Sungkyunkwan University, Seoul, Republic of Korea; ^5^Center for Health Promotion, Samsung Medical Center, Sungkyunkwan University School of Medicine, Seoul, Republic of Korea; ^6^Center for Cohort Studies, Total Healthcare Center, Kangbuk Samsung Hospital, Sungkyunkwan University School of Medicine, Seoul, Republic of Korea; ^7^Department of Health Sciences and Technology, SAIHST, Sungkyunkwan University, Seoul, Republic of Korea; ^8^Department of Intelligent Precision Healthcare Convergence, Sungkyunkwan University, Suwon, Republic of Korea

**Keywords:** NAFLD, amyloid-beta (Aβ), sex, Alzheimer’s disease, preclinical stage of Alzheimer’s disease

## Abstract

**Background:**

Non-alcoholic fatty liver disease (NAFLD) is known to be associated with a high risk of clinically diagnosed Alzheimer’s disease (AD). Additionally, the prevalence of NAFLD and AD is higher in elderly females than in males. However, a sex-specific association between NAFLD and amyloid-beta (Aβ) deposition remains unclear. Therefore, we investigated the sex-specific relationship between NAFLD and Aβ deposition in a large-sized cohort of cognitively unimpaired (CU) individuals.

**Methods:**

We enrolled 673 (410 [60.9%] females and 263 [39.1%] males) CU individuals aged ≥45 years who underwent Aβ positron emission tomography (PET). The presence of NAFLD, assessed using the hepatic steatosis index, and the severity of NAFLD, assessed using the Fibrosis-4 index, were considered predictors. Aβ deposition on PET was considered as an outcome.

**Results:**

Females had a higher frequency of NAFLD than males (48 and 23.2%, *p* < 0.001). Among females, the presence of NAFLD (β = 0.216, *p* < 0.001) was predictive of increased Aβ deposition, whereas among males, the presence of NAFLD (β = 0.191, *p* = 0.064) was not associated with Aβ deposition. Among females, the presence of NAFLD with low (β = 0.254, *p* = 0.039), intermediate (β = 0.201, *p* = 0.006), and high fibrosis (β = 0.257, *p* = 0.027) was predictive of increased Aβ deposition. Aβ deposition also increased as the severity of NAFLD increased in females (*p* for trend = 0.001).

**Conclusion:**

We highlight the marked influence of NAFLD and its severity on the risk of Aβ deposition in relation to sex. Furthermore, our findings suggest that sex-specific strategies regarding the management of NAFLD are necessary for the prevention of Aβ deposition.

## Introduction

Non-alcoholic fatty liver disease (NAFLD) is a highly prevalent chronic liver disease (Younossi et al., 2016) that has detrimental effects on multiple extrahepatic health conditions and liver-related outcomes. NAFLD is closely associated with metabolic syndromes, including diabetes and insulin resistance and complications of metabolic syndromes such as cardiovascular diseases (Gaggini et al., 2013). Thus, NAFLD is considered a hepatic manifestation of metabolic syndrome. Meanwhile, Alzheimer’s disease (AD) is characterized by the amyloid-beta (Aβ) deposition in the brain. Aβ deposition starts 15–20 years before the onset of cognitive impairment. Since Aβ positron emission tomography (PET) allowed us to detect Aβ deposition in living individuals, 15–30% of cognitively unimpaired (CU) individuals are found to have Aβ deposition (Sperling et al., 2020). Aβ deposition is also known to be associated with poor metabolic health (Gottesman et al., 2017). Specifically, metabolic syndromes, including diabetes, insulin resistance, hyperlipidemia, and increased weight variability, are closely related to Aβ deposition in CU individuals (Reed et al., 2014; Willette et al., 2015; Kang et al., 2022).

Many studies show that NAFLD is associated with a higher risk of clinically diagnosed AD (Shang et al., 2021; Kim et al., 2022). However, no previous studies have investigated the association between NAFLD and Aβ deposition in CU individuals. Aβ deposition in CU individuals represents the preclinical stage of AD (Shim and Morris, 2011). This preclinical stage has received considerable attention because early intervention may increase the possibility of therapeutic success. In addition, the prevention of Aβ deposition in CU individuals is regarded as primary prevention. Thus, investigation of the relationship between NAFLD and Aβ deposition in CU individuals has important implications for primary prevention, especially considering the paucity of known prevention methods.

Previous studies have suggested that there were differences in the effects of metabolic syndrome on brain health between females and males (Lee et al., 2018; Kim et al., 2019). In addition, the prevalence of NAFLD and AD was higher in elderly females than in elderly males (Alzheimer's Association, 2016; Lonardo et al., 2019; Tobari and Hashimoto, 2020). Therefore, the effects of NAFLD on Aβ deposition may differ depending on sex. However, the sex-specific relationship between NAFLD and Aβ deposition remains unclear.

In the present study, we aimed to investigate the sex-specific relationship between the presence of NAFLD, as defined using the hepatic steatosis index (HSI), and Aβ deposition in a large-sized cohort of CU individuals. Furthermore, we aimed to identify the sex-specific relationship between NAFLD severity, assessed using the Fibrosis-4 (FIB-4) index, and Aβ deposition. Given that brain health is more vulnerable to metabolic syndrome in females than in males, the effect of NAFLD on Aβ deposition may be more prominent in females than in males.

## Methods

### Study participants

We enrolled 673 cognitively unimpaired (CU) participants ≥45 years of age who underwent Aβ PET at the memory clinic in the Department of Neurology at Samsung Medical Center in Seoul, Korea, between August 2015 and November 2021. These participants comprised volunteers who applied for comprehensive dementia evaluation advertised in the local community, memory clinic, and paper; spouses of patients who visited the memory clinic; and participants with subjective cognitive decline. All participants underwent standardized neuropsychological test battery using the Seoul Neuropsychological Screening Battery 2nd edition (SNSB-II; Kang et al., 2021), brain magnetic resonance imaging (MRI), and laboratory tests, including liver function tests. All participants met the following criteria: (1) no medical history that was likely to affect cognitive function based on Christensen’s health screening criteria (Christensen et al., 1991); (2) no objective cognitive impairment in any cognitive domain on a comprehensive neuropsychological test battery (above at least −1.0 standard deviation (SD) of age-adjusted norms on any cognitive test); and (3) independence in activities of daily living. We excluded the participants with history of viral hepatitis or alcohol-liver disease, severe WMH (cap or band >10 mm and longest diameter of deep white matter lesion >25 mm), and structural lesions including cerebral infarction, intracranial hemorrhage, brain tumors, and hydrocephalus on MRI.

This study was approved by the Institutional Review Board of Samsung Medical Center approved. Written informed consent was obtained from all the participants.

### Fatty liver assessment

Fatty liver was defined using the HSI, which includes serum alanine aminotransferase (ALT, IU/L), serum aspartate aminotransferase (AST, IU/L), body mass index (BMI, kg/m^2^), sex, and presence of diabetes. The HSI was calculated using the following variables: HSI = 8 × (ALT/AST ratio) + BMI (+2, if female; +2, if diabetes mellitus).

Hepatic steatosis index has been created and validated for the detection of NAFLD in large populations, not only in Asians (Lee et al., 2010; Murayama et al., 2021) but also in Europeans (Meffert et al., 2014; Khani et al., 2022). In these validation studies in Asians, the area under the receiver operator characteristic curve was greater than 0.8. According to a validation study in a Korean population (Lee et al., 2010), fatty liver was defined as an HSI > 36 for the present study.

### Liver fibrosis assessment

Liver fibrosis was assessed using the Fibrosis-4 (FIB-4) index in participants with fatty liver. The FIB-4 index was calculated using the following formula: age (years) × AST (IU/L)/[platelet count (10^9^/L) × ALT (IU/L)^1/2^]. The FIB-4 index correlates highly with biopsy-proven advanced liver fibrosis (Lee et al., 2021). Furthermore, previous studies have demonstrated that the FIB-4 index was closely associated with liver disease-specific mortality (Unalp-Arida and Ruhl, 2017) and cardiovascular disease (Schonmann et al., 2021). Liver fibrosis was categorized according to the following cut-off values: ≤ 1.3, low fibrosis; > 1.3 and < 2.67, intermediate fibrosis; ≥ 2.67, high fibrosis.

### Aβ PET acquisition

All the participants underwent Aβ PET [^18^F-florbetaben (FBB) and ^18^F-flutemetamol (FMM)] PET using a Discovery STe PET/CT scanner (GE Medical Systems, Milwaukee, WI, United States). For FBB or FMM PET, a 20-min emission PET scan in dynamic mode (consisting of 4 × 5 min frames) was performed 90 min after the injection of a mean dose of 311.5 MBq FBB or 197.7 MBq FMM, respectively. Three-dimensional PET images were reconstructed in a 128 × 128 × 48 matrix with 2 mm × 2 mm × 3.27 mm voxel size using the ordered-subsets expectation maximization algorithm (FBB, iteration = 4 and subset = 20; FMM, iteration = 4 and subset = 20).

### Aβ PET quantification using Centiloid values

Amyloid-beta uptake was defined according to Aβ PET quantification using Centiloid values. We performed a direct comparison of FBB-FMM Centiloid (dcCL) method previously developed by our group (Cho et al., 2020) to standardize the quantification of Aβ PET images obtained using different ligands. The dcCL method for FBB and FMM PET enables the transformation of the standardized uptake value ratio (SUVR) of FBB and FMM PETs to dcCL values directly without conversion to the ^11^C-labeled Pittsburgh compound SUVR.

There are three steps for obtaining dcCL values (Cho et al., 2020): (1) preprocessing of PET images, (2) determination of the global cortical target volume of interest (CTX VOI), and (3) conversion of dcSUVR to dcCL values. First, to preprocess the Aβ PET images, PET images were co-registered to each participant’s MR image and then normalized to a T1-weighted MNI-152 template using the SPM8 unified segmentation method. We used T1-weighted MR image correction with the N3 algorithm only for intensity non-uniformities, without applying corrections to the PET images for brain atrophy or partial volume effects. Second, we used the FBB-FMM CTX VOI, defined as the area of AD-specific brain Aβ deposition in our previous study (Cho et al., 2020). Briefly, to exclude areas of aging-related brain Aβ deposition, the FBB-FMM CTX VOI was generated by comparing SUVR parametric images (with the whole cerebellum as a reference area) between 20 typical patients with Alzheimer’s disease-related cognitive impairment (AD-CTX) and 16 healthy elderly participants (EH-CTX) who underwent both FBB and FMM PET scans. To generate the FBB-FMM CTX VOI, the average EH-CTX image was subtracted from the average AD-CTX image. We then defined the FBB-FMM CTX VOI as the area of AD-related brain Aβ accumulation common to both FBB and FMM PET. Finally, the dcSUVR values of the FBB-FMM CTX VOI were converted into dcCL units using the dcCL conversion equation. The dcCL equation was derived from the FBB-FMM CTX VOI separately for FBB and FMM PET and applied to FBB and FMM dcSUVR.

To determine the participants’ dcCL cut-off-based Aβ positivity, we applied the optimal cut-off value derived using *k*-means cluster analysis in 527 independent samples of participants with normal cognition. The cut-off value was set at 27.08, representing the 95th percentile of the lower cluster, and the whole cerebellum was used as a reference region.

**Figure 1:**
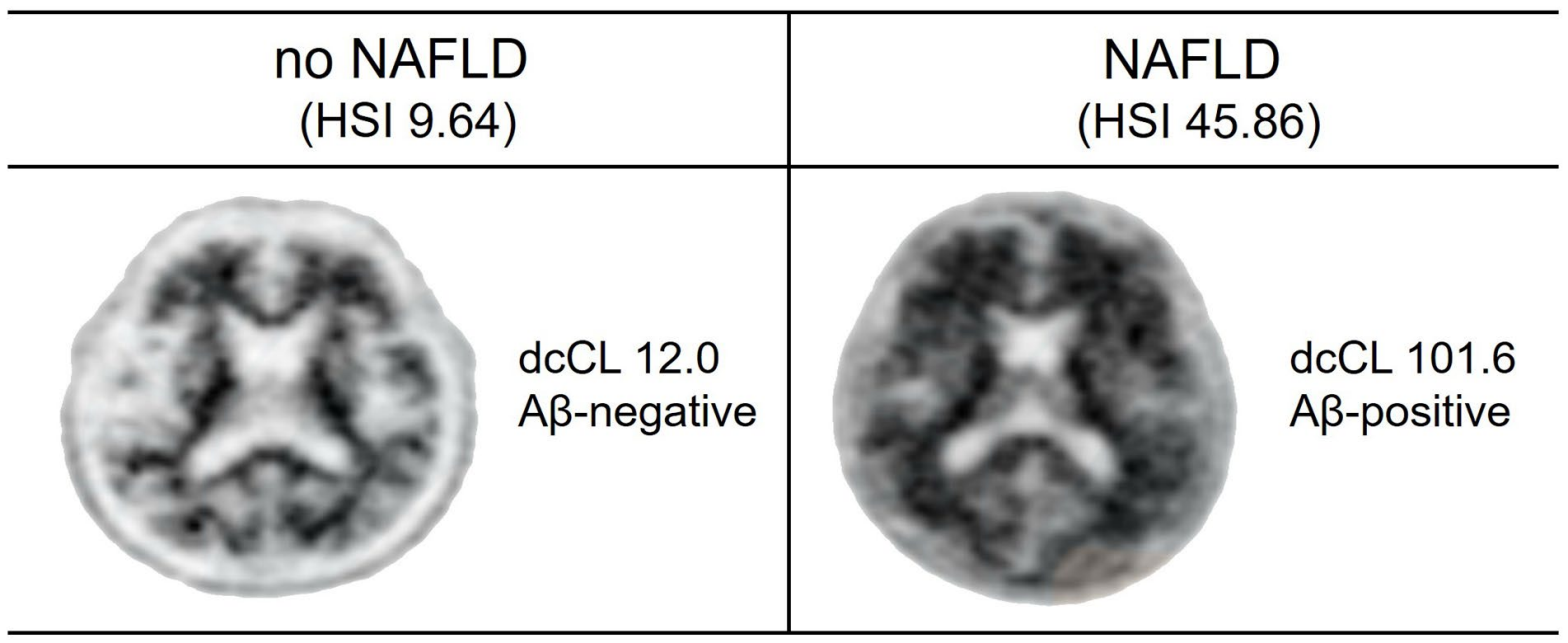
Aβ uptake and positivity in study participants. Two representative cases of Aβ PET are shown. One participant without NAFLD has low Aβ uptake (dcCL, 12.0) and Aβ-negative on PET scan. Another participant with NAFLD has high Aβ uptake (dcCL, 101.6) and Aβ-positive on PET scan.

Figure 1 presents two representative cases with respect to Aβ uptake and Aβ positivity on PET scan.

### Standardized neuropsychological test battery

All participants underwent the SNSB-II (Kang et al., 2021), which includes standardized and validated tests of various cognitive functions. The SNSB-II evaluates many cognitive factors, including verbal and visual memory, visuo-constructive function, language, praxis, components of Gerstmann syndrome (acalculia, agraphia, right/left disorientation, and finger agnosia), and frontal/executive functions. We chose to use six cognitive measures, which are representative and important neuropsychological tests, to evaluate cognitive function in five cognitive domains as follows: (1) Memory: Seoul Verbal Learning Test and Rey–Osterrieth Complex Figure Test (RCFT) delayed recall; (2) Language: Korean version of the Boston Naming Test; (3) Visuospatial function: RCFT copy; (4) Frontal executive function: Stroop Test color reading; and (5) Attention: Digit Span Test backward.

### Statistical analyses

All statistical analyses were performed separately in males and females. Independent *t*-tests and chi-square tests were used to compare the demographic and clinical characteristics of the participants.

To investigate the association between the presence of NAFLD and Aβ deposition, we performed linear regression analyses in males and females, with the presence of NAFLD as a predictor, and quantified dcCL values as an outcome after controlling for age, education years, hypertension, diabetes, hyperlipidemia, BMI, and Apolipoprotein E4 (*APOE4*) genotype. To investigate the association between the severity of NAFLD and Aβ deposition, we performed linear regression analyses in males and females, with the severity of NAFLD (no NAFLD, NAFLD with low fibrosis, NAFLD with intermediate fibrosis, and NAFLD with high fibrosis) as a predictor and quantified dcCL values as an outcome, after controlling for age, education years, hypertension, diabetes, hyperlipidemia, BMI, and *APOE4* genotype. We also performed a linear trend test using linear regression analysis in males and females, with the severity of NAFLD as a continuous variable, after controlling for age, education years, hypertension, diabetes, hyperlipidemia, BMI, and *APOE4* genotype. To evaluate the interaction of sex and NAFLD on Aβ deposition, we performed a linear regression analysis, with NAFLD and sex together as the main effect and NAFLD × sex as an interaction effect, after controlling for age, education years, hypertension, diabetes, hyperlipidemia, BMI, and *APOE4* genotype in all participants.

A sensitivity analysis using cut-off-based categorization rather than quantified dcCL values was performed to further validate the relationship between NAFLD and Aβ deposition. We used logistic regression analysis with NAFLD as a predictor and Aβ positivity as an outcome after controlling for age, education years, hypertension, diabetes, hyperlipidemia, BMI, and *APOE4* genotype.

All reported *p* values were two-sided, and the significance level was set at 0.05. All the analyses were performed using SAS version 9.4 (SAS Institute Inc., Cary, NC, United States).

## Results

### Clinical characteristics of participants

Among the 673 participants, 410 were females and 263 were males (Table 1). Females had a lower mean age (70.7 ± 7.7 and 71.8 ± 7.2, *p* = 0.049), education years (10.9 ± 4.7 and 13.8 ± 3.9, *p* < 0.001), and BMI (23.8 ± 3.1 and 24.7 ± 2.6, *p* < 0.001) than males. There were no differences between males and females in the mean dcCL values (19.29 ± 30.68 and 23.73 ± 38.74, *p* = 0.117), the frequency of Aβ + (24.9 and 26.6%, *p* = 0.679), and the frequency of *APOE4* genotype (25.1 and 26.6%, *p* = 0.732). Females had a lower frequency of hypertension (43.4 and 52.5%, *p* = 0.027) and diabetes (15.4 and 25.1%, *p* = 0.002) than males. However, the frequency of NAFLD was higher in females than in males (48 vs. 23.2%, *p* < 0.001).

**Table 1:** Demographics and clinical information of participants.

	Females (*n* = 410)	Males (*n* = 263)	*p* value
*Demographics*			
Age, years	70.7 ± 7.7	71.8 ± 7.2	0.049
Education, years	10.9 ± 4.7	13.8 ± 3.9	< 0.001
*APOE4* genotype	103 (25.1%)	70 (26.6%)	0.732
Hypertension	178 (43.4%)	138 (52.5%)	0.027
Diabetes	63 (15.4%)	66 (25.1%)	0.002
Hyperlipidemia	202 (49.3%)	121 (46.0%)	0.455
BMI	23.8 ± 3.1	24.7 ± 2.6	< 0.001
*Liver profiles*			
Presence of NAFLD	197 (48.0%)	61 (23.2%)	< 0.001
NAFLD severity			< 0.001
Low fibrosis	27 (6.6%)	2 (0.8%)	
Intermediate fibrosis	140 (34.1%)	44 (16.7%)	
High fibrosis	30 (7.3%)	15 (5.7%)	
*Aβ deposition*			
dcCL values	19.3 ± 30.7	23.7 ± 38.7	0.117
^*^Aβ positivity	102 (24.9%)	70 (26.6%)	0.679

Values are presented as mean ± standard deviation. ^*^Aβ positivity was determined using optimal cut-off values of dcCL. Aβ, Amyloid-beta; *n*, Number of patients whose data were available for analysis; APOE4, Apolipoprotein E4; BMI, Body mass index; dcCL, Direct comparison of FBB-FMM Centiloid; and NAFLD, non-alcoholic fatty liver disease.

### Effects of NAFLD on Aβ deposition

**Figure 2:**
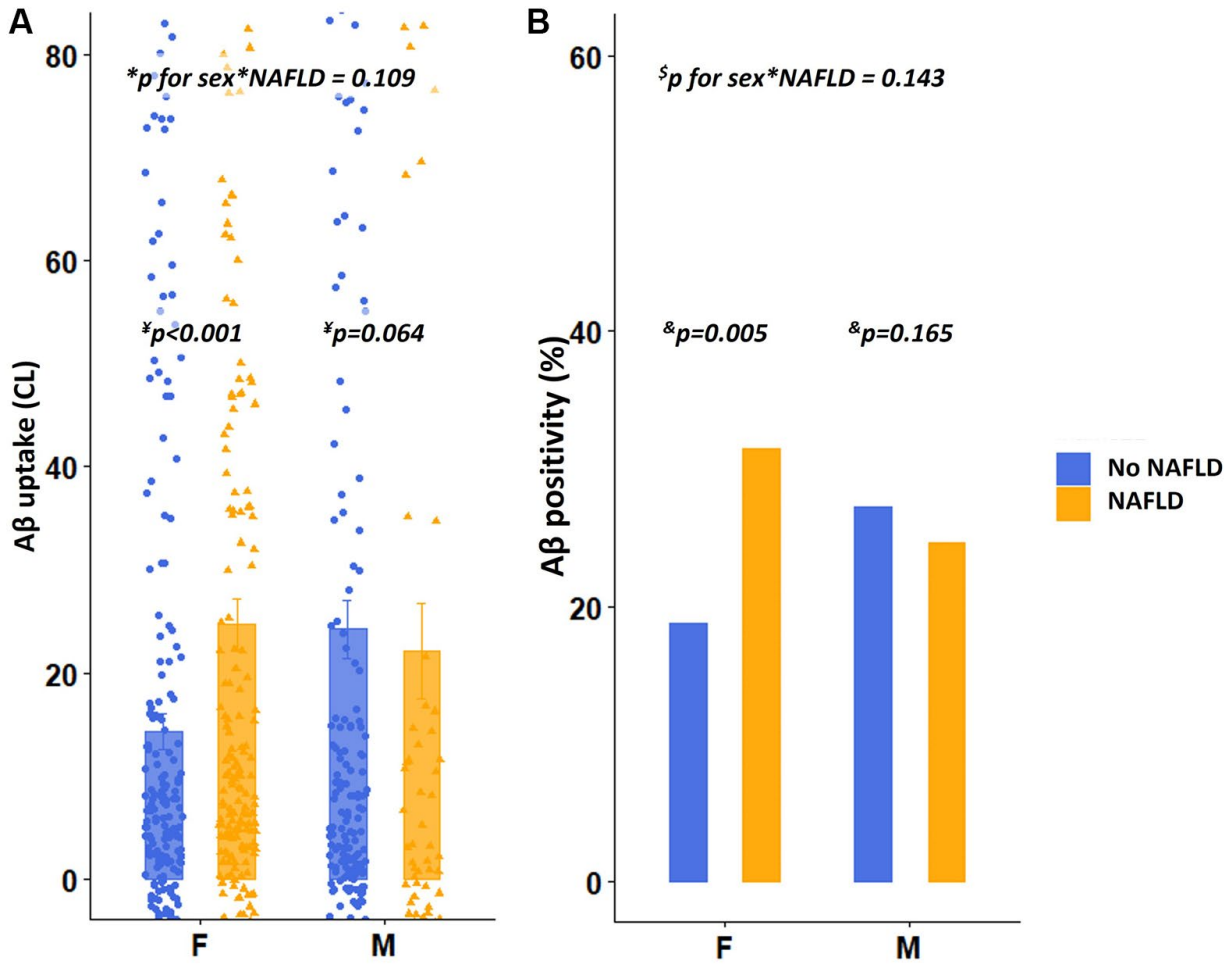
Differences in Aβ uptake and positivity according to the presence of NAFLD. **(A)** Values depicted in the bar plot represent the presence of NAFLD on the X-axis and Aβ uptake (CL) on the Y-axis. **(B)** Values depicted in the bar plot represent the presence of NAFLD on the X-axis and Aβ positivity (%) on the Y-axis. ^*^p for interaction was estimated using the linear regression analyses, including the presence of NAFLD as the main effect and sex × presence of NAFLD as an interaction effect after controlling for age, education years, hypertension, diabetes, hyperlipidemia, BMI, and *APOE4* genotype. ^¥^*p* value was estimated using linear regression analyses, with the presence of NAFLD as a predictor, after controlling for age, education years, hypertension, diabetes, hyperlipidemia, BMI, and *APOE4* genotype. ^$^*p* for interaction was estimated using logistic regression analyses, including the presence of NAFLD as a main effect and sex × severity of NAFLD as an interaction effect after controlling for age, education years, and *APOE4* genotype. ^&^*p* value was estimated using logistic regression analyses, with the severity of NAFLD as a predictor after controlling for age, education years, hypertension, diabetes, hyperlipidemia, BMI, and *APOE4* genotype. Aβ, Amyloid; *APOE4*, Apolipoprotein E4; BMI, Body mass index; dcCL, Direct comparison of FBB-FMM Centiloid; and NAFLD, Non-alcoholic fatty liver disease.

As illustrated in Figure 2A, among females, the presence of NAFLD (β = 0.216, *p* < 0.001) was predictive of increased dcCL (Table 2). However, among males, the presence of NAFLD (β = 0.191, *p* = 0.064) was not associated with dcCL values (Table 2). There was no interaction effect between sex and the presence of NAFLD (sex × NAFLD, *p* = 0.109) on dcCL values.

**Table 2:** Relationship between NAFLD and Aβ deposition.

		Females		Males	
		^*^Beta (SE)	*p*	^*^Beta (SE)	*p*
Presence	NAFLD	0.216 (0.060)	< 0.001	0.191 (0.103)	0.064
^¥^Severity	Low fibrosis	0.254 (0.123)	0.039	0.139 (0.440)	0.917
	Intermediate fibrosis	0.201 (0.064)	0.006	0.262 (0.117)	0.077
	High fibrosis	0.257 (0.108)	0.027	0.018 (0.170)	0.917

^*^Beta was obtained using linear regression analyses, with the presence or severity of NAFLD separately as a predictor, after controlling for age, education years, hypertension, diabetes, hyperlipidemia, BMI, and APOE4 genotype. ^¥^*p* values were obtained after FDR correction. Aβ, amyloid; APOE4, Apolipoprotein E4; BMI, body mass index; FDR, false discovery rate; NAFLD, Non-alcoholic fatty liver disease; and SE, standard error.

**Figure 3:**
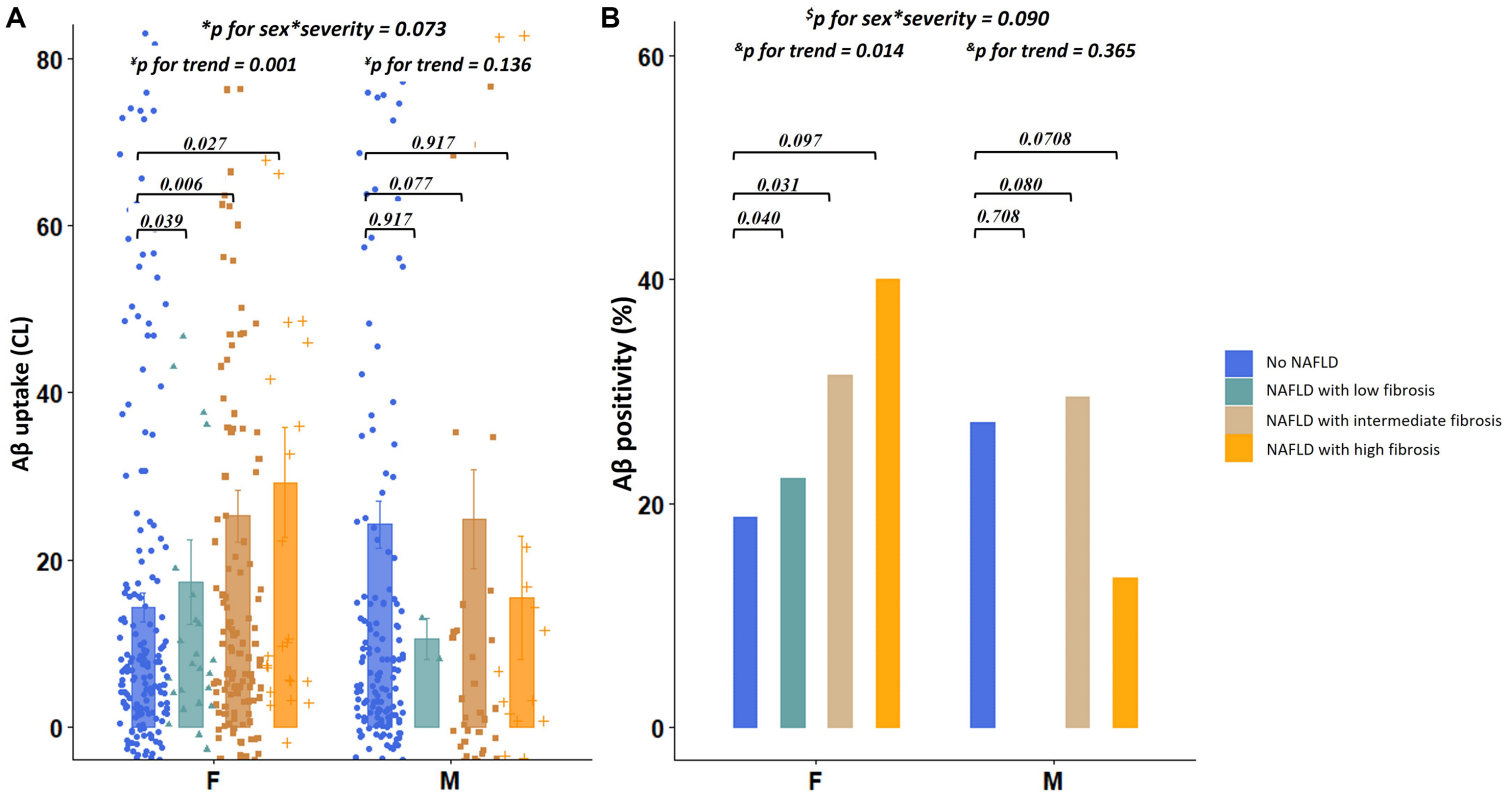
Differences in Aβ uptake and positivity according to the severity of NAFLD. **(A)** Values depicted in the bar plot represent the severity of NAFLD (no NAFDL, NAFLD with low fibrosis, NAFLD with intermediate fibrosis, and NAFLD with high fibrosis) on the X-axis and Aβ uptake (CL) on the Y-axis. **(B)** Values depicted in the bar plot represent the severity of NAFLD (no NAFDL, NAFLD with low fibrosis, NAFLD with intermediate fibrosis, and NAFLD with high fibrosis) on the X-axis and Aβ positivity (%) on the Y-axis. ^*^p for interaction was estimated using linear regression analyses, including the severity of NAFLD as the main effect and sex × severity of NAFLD as an interaction effect after controlling for age, education years, hypertension, diabetes, hyperlipidemia, BMI, and *APOE4* genotype. ^¥^*p* for trend was estimated using linear regression analyses, with the severity of NAFLD as a continuous variable after controlling for age, education years, hypertension, diabetes, hyperlipidemia, BMI, and *APOE4* genotype. ^$^*p* for interaction was estimated using logistic regression analyses, including the presence of NAFLD as the main effect and sex × severity of NAFLD as an interaction effect after controlling for age, education years, hypertension, diabetes, hyperlipidemia, BMI, and *APOE4* genotype. ^&^*p* for trend was estimated using logistic regression analyses, with the severity of NAFLD as a continuous variable after controlling for age, education years, hypertension, diabetes, hyperlipidemia, BMI, and *APOE4* genotype. Aβ, Amyloid; *APOE4*, Apolipoprotein E4; BMI, Body mass index; dcCL, Direct comparison of ^18^F-florbetaben-^18^F-flutemetamol Centiloid; and NAFLD, Non-alcoholic fatty liver disease.

Regarding the severity of NAFLD, among females, the presence of NAFLD with low (β = 0.254, *p* = 0.039), intermediate (β = 0.201, *p* = 0.006), and high fibrosis (β = 0.257, *p* = 0.027) was predictive of increased dcCL values (Table 2; Figure 3A). However, among males, there were no differences in the dcCL values between those without and with NAFLD with low (β = 0.139, *p* = 0.917), intermediate (β = 0.262, *p* = 0.077), or high fibrosis (β = 0.018, *p* = 0.917; Table 2).

In the linear trend test, as illustrated in Figure 3A, the dcCL values increased as the severity of NAFLD (low, intermediate, and high fibrosis) increased in females (*p* for trend = 0.001), which was not the case with the males (*p* for trend = 0.136). There was no interaction effect between sex and NAFLD severity (sex × severity of NAFLD, *p* = 0.073) on dcCL values.

### Sensitivity analysis

Regarding the categorical values of Aβ, among females, presence of NAFLD [odds ratio (OR) = 2.32, *p* = 0.005] was independently associated with higher Aβ positivity (Figure 2B), whereas, among males, the presence of NAFLD (OR = 1.83, *p* = 0.165) was not associated with Aβ positivity (Figure 2B). There was no interaction effect between sex and the presence of NAFLD (sex × NAFLD, *p* = 0.143) on Aβ positivity. The relationship between the severity of NAFLD and Aβ positivity showed a similar trend. Aβ positivity became higher as the severity of NAFLD (low, intermediate, and high fibrosis) increased in females (*p* for trend = 0.014), whereas Aβ positivity was not associated with the severity of NAFLD in males (*p* for trend = 0.365, Figure 3B). There was no interaction effect between sex and NAFLD severity (sex × NAFLD severity, *p* = 0.090) on Aβ positivity.

## Discussion

In the present study, we systematically investigated the sex-specific relationships between NAFLD and Aβ deposition in a large number of CU individuals. We found that the presence of NAFLD, as defined using HSI, was associated with higher Aβ deposition in females but not in males. The severity of NAFLD, as defined using FIB-4, was also associated with higher Aβ deposition in females but not in males. Thus, our findings suggest that there may be a sex-specific relationship between NAFLD and Aβ deposition. Therefore, our results may contribute to the design of sex-specific strategies for NAFLD management to prevent Aβ deposition in CU individuals.

In the present study, females had a higher incidence of NAFLD than males. Considering our female participants to be in postmenopausal status, our findings are consistent with previous studies showing that the frequency of NAFLD is higher in postmenopausal females than in males (Lonardo et al., 2019; Tobari and Hashimoto, 2020). However, the frequency of NAFLD is higher in males, when compared to females of pre-menopausal age group (Lonardo et al., 2019; Tobari and Hashimoto, 2020). This difference in the results before and after menopause could be explained by the influence of estrogen. Estrogen promotes well-metabolic conditions by regulating energy homeostasis, enhancing insulin release, modulating the role of growth hormones, and preventing inflammation (Rettberg et al., 2014). Hormone therapy has been reported to have a protective effect against NAFLD in postmenopausal females (McKenzie et al., 2006).

Our first major finding was that NAFLD was associated with higher Aβ deposition in females. Previous studies have demonstrated that NAFLD is closely related to a greater risk of cognitive impairment and the clinical diagnosis of AD in the elderly population (Seo et al., 2016; Jeong et al., 2022). However, to the best of our knowledge, the association between NAFLD and Aβ deposition on PET has not been found in CU individuals. Several mechanisms may explain the association between NAFLD and Aβ deposition. First, NAFLD-related chronic inflammation may contribute to the activation of microglial cells in the brain, leading to increased levels of inflammatory cytokines, eventually resulting in increased Aβ deposition. An animal study found that NAFLD-induced high-fat diet aggravates neuroinflammation, accompanied by increased Aβ deposition (Kim et al., 2016). Additionally, another study suggested that increased lipocalin-2 levels related to NAFLD may induce the breakdown of the blood–brain barrier and increase the levels of inflammatory cytokines in the brain (Mondal et al., 2020). Alternatively, the low expression of low-density lipoprotein receptor-related protein (LRP-1) observed in patients with NAFLD may lead to decreased Aβ clearance (Tamaki et al., 2007). The stimulation of LRP-1 expression in the hepatocyte decreases Aβ deposition in the brain of AD mice model, which improves cognitive function (Sehgal et al., 2012).

An intriguing finding of the present study is that the relationship between NAFLD and Aβ deposition is valid in females but not in males. Although the exact mechanism remains uncertain, multifactorial factors, including sex hormones and socio-behaviors, may underlie the sex-specific relationship between NAFLD and Aβ deposition. Estrogen deficiency in postmenopausal females with NAFLD may be a risk factor for Aβ deposition. Estrogen may also protect from Aβ deposition through anti-inflammatory properties and neurotrophic effects (Morinaga et al., 2007; Anderson et al., 2017). Furthermore, estrogen deficiencies may be attributed to blood–brain barrier breakdown, suggesting that postmenopausal females are more vulnerable to a highly systemic-inflammatory state (Maggioli et al., 2016). However, further comprehensive studies are necessary to identify the exact mechanism underlying the interactions between sex, NAFLD, Aβ deposition, and inflammation.

Our second major finding was that the severity of NAFLD might be associated with Aβ deposition. In line with our findings, recent studies have shown that advanced liver fibrosis, which determines the severity of NAFLD, is predictive of cognitive decline and development (Weinstein et al., 2019; Solfrizzi et al., 2020). A link between NAFLD severity and Aβ deposition may occur through systemic inflammation. The interleukin signaling pathway is an important shared pathomechanism between NAFLD and AD (Karbalaei et al., 2018). Furthermore, animal studies have reported that systemic inflammation precipitates Aβ deposition, which eventually causes AD (Krstic et al., 2012; Carret-Rebillat et al., 2015).

The strength of the present study is that we systematically investigated the effects of NAFLD on Aβ deposition in a large-sized cohort of CU individuals. Especially, enrollment of CU individuals ameliorated the potential reverse effects of dementia on NAFLD. However, this study has several limitations that should be addressed. First, we could not assess NAFLD using imaging or histological confirmation. However, this argument is mitigated to some degree, considering that HSI is a widely used surrogate marker of NAFLD and is well-validated (Lee et al., 2010; Meffert et al., 2014; Murayama et al., 2021; Khani et al., 2022). Second, since this was a cross-sectional study, it was difficult to guarantee the causality of Aβ deposition due to NAFLD. Third, although Aβ deposition is a necessary but not sufficient condition for AD, we did not assess the other AD biomarkers including phosphorylated tau. Finally, our participants were mainly recruited from individuals who underwent comprehensive dementia evaluation in the memory clinic. This study may have resulted in the enrollment of healthier or more “health-seeking” individuals, which may also limit the generalizability of this study to other community-based individuals. Nevertheless, our study is noteworthy, suggesting that the prevention and management of NAFLD might be important for the primary prevention of Aβ deposition in CU individuals. Furthermore, CU females were more vulnerable to developing NAFLD and had more prominent effects of NAFLD on Aβ deposition than males. Thus, screening for early detection of NAFLD is necessary to identify individuals at high risk of Aβ deposition, especially females.

In conclusion, we highlighted that the presence of NAFLD was predictive of a higher Aβ burden in females but not in males. The severity of NAFLD is also associated with a higher Aβ burden in females. Therefore, our results suggest that sex-specific strategies are required for NAFLD management to prevent Aβ deposition in CU individuals.

## Data availability statement

The raw data supporting the conclusions of this article will be made available by the authors, without undue reservation.

## Ethics statement

This study was approved by the Institutional Review Board of Samsung Medical Center. The studies were conducted in accordance with the local legislation and institutional requirements. The participants provided their written informed consent to participate in this study.

## Author contributions

SK: Conceptualization, Data curation, Formal analysis, Funding acquisition, Investigation, Methodology, Writing – original draft, Writing – review & editing. HY: Formal analysis, Writing – review & editing. BC: Data curation, Writing – review & editing. JK: Data curation, Writing – review & editing. HJ: Data curation, Writing – review & editing. HK: Data curation, Writing – review & editing. MK: Data curation, Writing – review & editing. KO: Data curation, Writing – review & editing. S-BK: Data curation, Writing – review & editing. DN: Data curation, Writing – review & editing. YC: Data curation, Writing – review & editing. SS: Conceptualization, Funding acquisition, Investigation, Methodology, Supervision, Writing – review & editing.
